# Modelling the Effect of COVID-19 Lockdown on Air Pollution in Makkah Saudi Arabia with a Supervised Machine Learning Approach

**DOI:** 10.3390/toxics10050225

**Published:** 2022-04-29

**Authors:** Turki M. Habeebullah, Said Munir, Jahan Zeb, Essam A. Morsy

**Affiliations:** 1 Department of Environmental and Health Research, The Custodian of the Two Holy Mosques Institute for Hajj and Umrah Research, Umm Al Qura University, Makkah 24382, Saudi Arabia; tmhabeebullah@uqu.edu.sa (T.M.H.); jzhabib@uqu.edu.sa (J.Z.); eamibrahim@uqu.edu.sa (E.A.M.); 2 Institute for Transport Studies, Faculty of Environment, University of Leeds, Leeds LS2 9JT, UK

**Keywords:** COVID-19 lockdown, air quality, Makkah, NO_2_, O_3_, PM_10_, intervention, machine learning

## Abstract

To reduce the spread of COVID-19, lockdowns were implemented in almost every single country in the world including Saudi Arabia. In this paper, the effect of COVID-19 lockdown on O_3_, NO_2_, and PM_10_ in Makkah was analysed using air quality and meteorology data from five sites. Two approaches were employed: (a) comparing raw measured concentrations for the lockdown period in 2019 and 2020; and (b) comparing weather-corrected concentrations estimated by the machine learning approach with observed concentrations during the lockdown period. According to the first approach, the average levels of PM_10_ and NO_2_ decreased by 12% and 58.66%, respectively, whereas the levels of O_3_ increased by 68.67%. According to the second approach, O_3_ levels increased by 21.96%, while the levels of NO_2_ and PM_10_ decreased by 13.40% and 9.66%, respectively. The machine learning approach after removing the effect of changes in weather conditions demonstrated relatively less reductions in the levels of NO_2_ and PM_10_ and a smaller increase in the levels of O_3_. This showed the importance of adjusting air pollutant levels for meteorological conditions. O_3_ levels increased due to its inverse correlation with NO_2_, which decreased during the lockdown period.

## 1. Introduction

In December 2019, Coronavirus Disease 2019 (COVID-19) was reported from Wuhan, China, and spread quickly to the rest of the world [1]. To curtail the spread of COVID-19, lockdowns were announced in different countries worldwide, and COVID-19 was officially announced as a pandemic by WHO [2]. In April 2022, WHO confirmed over 500 million victims of COVID-19, with over 6.2 million mortalities [3]. All sectors of life, including scientists, medical doctors, decision makers, and establishments worked together for the alleviation of COVID-19 [4]. The implemented lockdown was helpful to control the transmission of the virus but equally, it significantly affected the global economies [5,6]. In addition to curtailing the transmission of the virus, the lockdown resulted in clean air as reported by a number of researchers in many countries, e.g., [7,8,9,10]. Dutheil et al. [7] reported a reduction in NO_2_ concentrations measured with the TROPOMI sensor on-board ESA’s Sentinel-5 satellite before and after the COVID-19 pandemic. Sharma et al. [8] analysed the concentrations of several pollutants, namely, PM_10_, PM_2.5_, CO, and NO_2_ during the lockdown period in India and reported about 43, 31, 10, and 18% reductions, respectively, compared to previous years. Kroll et al. [9] argued that COVID-19 lockdown not only affected emissions but also the chemical transformations of pollutants in the atmosphere. Munir et al. [10] reported reductions in several pollutants and emphasised that the approach used for quantifying the reduction was vital. However, the clean air episode was not permanent and sustainable because of its unbearable socioeconomic cost [4]. The improved air quality helped avoid approximately 50,000 pollution-related mortalities during the pandemic months [11], which showed the importance of clean air in urban areas. On the other hand, poor air quality is reported to be associated with 4.2 million deaths globally [12]. 

In India, several studies were conducted in different cities during the lockdown period and showed an improvement in air quality and a reduction in the concentrations of PM_10_ by 58%, PM_2.5_ by 73.85%, SO_2_ by 58%, NH_3_ by 75%, CO by 60%, and NO_2_ by 79% [13,14]. Anil and Alagha [15] conducted air quality monitoring at eight stations in the eastern province of Saudi Arabia from September 2019 to July 2020. NO_2_ concentrations were reduced by 12–86% during lockdown and by 14–81% in the post-lockdown period. Concentrations of CO were reduced by 5.8–55%, PM_10_ by 9–30%, and SO_2_ by 21–70% during the lockdown period. Only O_3_ concentrations increased during lockdown (6.3–45%) and in the post-lockdown (18–263%) period. Kumari and Toshniwal [16] conducted a worldwide study in twelve cities and reported a decrease in the concentrations of PM_2.5_, PM_10_, and NO_2_ ranging from 20–64%. In Ecuador, the maximum reduction reported in NO_2_ concentration was 23% [17]. A 12% global reduction in PM_2.5_ concentration during the lockdown period was reported by Rodríguez-Urrego and Rodríguez-Urrego [18]. Another study concluded a 30% decrease in the overall pollution level, particularly in some COVID-19 axis areas such as the USA, Italy, Spain, and Wuhan [19]. Munir et al. [5] analysed the effect of COVID-19 lockdown on NO, NO_2_, NO_x_, PM_2.5_, and PM_10_ in northern England, UK and reported a reduction of 43.31 to 69.75% for NO, 37.13 to 55.54% for NO_2_, 41.52 to 63.99% for NO_x_, 29.93 to 40.26% for PM_2.5_, and 2.36 to 19.02% for PM_10_. Morsy et al. [20] monitored the concentration of air pollutants in the holy city of Makkah, Saudi Arabia, during the lockdown period and compared the results with the pre-lockdown period. Considerable reductions were observed in SO_2_, NO_2_, CO, O_3_, and PM_10_, i.e., 26.34%, 28.99%, 26.24%, 11.62%, and 30.03%, respectively, induced by the strict lockdown measures. Klara and Maria [21] measured air quality in Portugal using 68 monitoring stations and covered rural, urban, and suburban zones. This study reported a 15–71% and 10–70% reduction in traffic-related NO_2_ and PM, respectively. A 35% reduction in SO_2_ was observed due to the suspension of industrial activities. 

On 2 March 2020, the first positive case of the coronavirus was reported in the Kingdom of Saudi Arabia. Initially, three major steps were taken by the Kingdom of Saudi Arabia to minimise the spread of the virus: the deferral of the Umrah visit (4 March 2020), closing the educational institutions (8 March 2020), and stopping worldwide flight operations (9 March 2020). Using these measures, the Kingdom succeeded to slow down the spread of the pandemic but could not completely stop the spread; therefore, on 23 March 2020, a partial lockdown was enforced and on 25 March 2020, inter-provincial transportation was seized. Finally, a complete lockdown was enforced in the country on 6 April 2020. The lockdown was relaxed on 31 May 2020 in Makkah and partial activities in mosques, restaurants, malls, parks, and cafes were allowed [15,20]. Such lockdowns were implemented in many countries around the world, which resulted in improved environmental conditions including air quality. 

Makkah city hosts almost 10 million visitors of Umrah annually. The visitors for Umrah are spread over the whole year but are more concentrated in the holy month of Ramadan (9th month of the Islamic calendar). Another 3 million (approx.) visitors come to perform the pilgrimage (Hajj) every year. The Hajj event takes place for 10 to 15 days in the 12th month of the Islamic calendar. Furthermore, the population of Makkah has been increasing rapidly and the city has been spreading out. Road traffic used for the transportation of both residents and visitors is the key source of pollutant emissions that compromise the air quality of Makkah. During the lockdown period, the number of visitors was limited even during the holy month of Ramadan and Hajj. Visitors to Makkah were only allowed from inside the Kingdom [20]. 

In this paper, the effect of COVID-19 lockdown on air quality was analysed using air quality data from five monitoring stations in Makkah using parallel and machine learning approaches [10], focusing on three major pollutants, namely, NO_2_, O_3_, and PM_10_. These pollutants are the most important from the public health point of view as they can cause various health problems [22,23]. The main aim was to quantify the effect of lockdown on air quality and present an improved methodology for assessing the impact of the interventions compared to the previous studies conducted in the Kingdom of Saudi Arabia [15,20], which employed only a sequential approach as described by Munir et al. [10]. The uniqueness of this study is that, in contrast to a previous study using a sequential approach [10], it applied a parallel approach using measured concentrations. Furthermore, the effect was analysed using weather-corrected (meteorologically adjusted) data. Previous studies carried out in Makkah have not deweathered the data and have simply compared pre-lockdown and lockdown periods using raw data.

## 2. Materials and Methods

**Figure 1:**
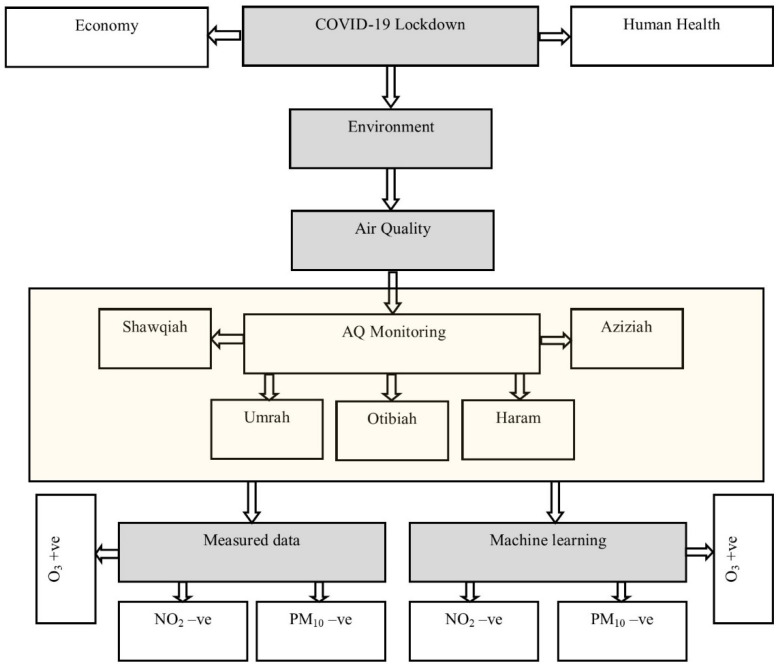
Schematic diagram of the processes, methodology, and outcomes.

The basic research question was how COVID-19 lockdown affected air quality in hot and dry climatic conditions and what method should be used to extract the impact of the intervention. To answer this question, in this paper, the effect of the COVID-19 lockdown on air pollution in Makkah, Saudi Arabia was assessed by employing a supervised machine learning approach [10,24]. Air pollutants and meteorology data were used from five monitoring stations. Firstly, air pollutants levels were compared during the lockdown months in 2019 with 2020 using measured data, and secondly, pollutant levels were predicted for the lockdown months using a generalised additive model (GAM) and compared with the measured concentrations, as described in the following sections. Figure 1 shows the schematic diagram of the whole procedure followed in this paper. 

### 2.1. Air Quality Monitoring Stations

**Figure 2:**
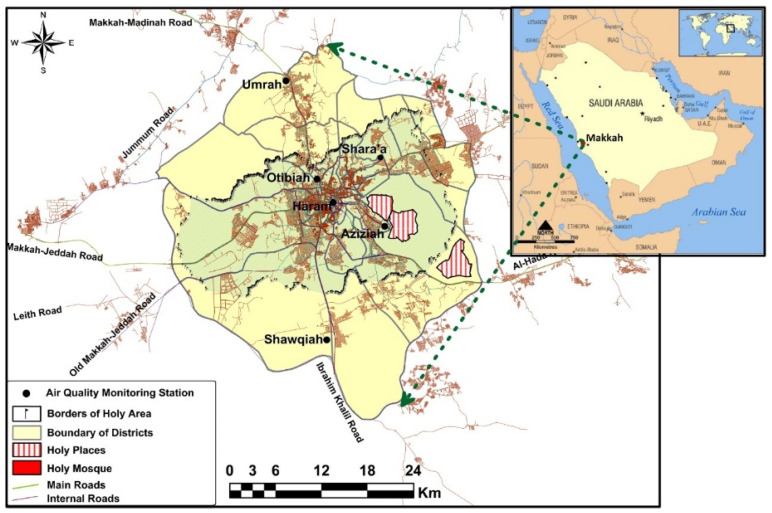

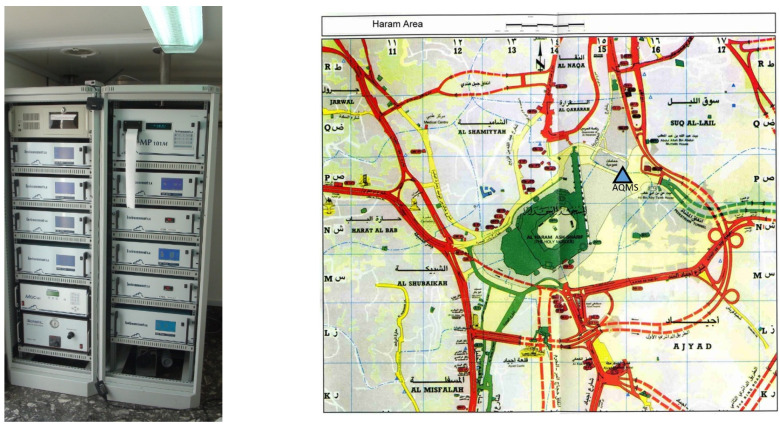
Map showing the location of monitoring sites in Makkah: Umrah, Otibiah, Aziziah, Shawqiah, and Haram. No data were available from Shara’a for 2020; therefore, comparison was not made for this site. The lower panel shows the location of the Haram site inside the Holy Mosque and the equipment (Environment SA) used for collecting the data.

Hourly measured data of several air pollutants and meteorological variables for years 2019 and 2020 were collected at five air quality monitoring stations in Makkah, Saudi Arabia (Figure 2). The air quality monitoring stations were: Aziziah, Otibiah, Shawqiah, Haram, and Umrah. The Aziziah site (21.40377, 39.87837) is close to the Holy Mosque and is considered an urban traffic site. Aziziah district is famous for its busy roads and the presence of many commercial centres including many shopping malls and restaurants. The Shawqiah site (21.36589, 39.80747) is located in the southern part of Makkah city and surrounded by an intensive residential complex. Shawqiah district is considered one of the main residential districts of Makkah, belonging to the municipality of Al-Shawqiah. Otibiah station (21.44222, 39.81189) experiences highly commercial activities due to its closeness to the centre of Makkah city. Haram station (21.42464, 39.82917) is located in the eastern yard of the Grand Holy Mosque (Figure 2) and can be considered an urban background site. Finally, Umrah station (21.50847, 39.79339) is located on the north-western side of Makkah city. Umrah district is the industrial city of Makkah. Umrah district has the main electrical power station of Makkah, which is responsible for fulfilling the electrical and power need of the city of Makkah.

The sensors used for the measurement of the pollutants on these sites are based on automatic continuous detection techniques: SO_2_ (APSA370, UV fluorescence); NO and NO_2_ (APNA370, chemiluminescence); CO (APMA370, IR Absorption); PM_10_ (BAM1020, bray); and O_3_ (APOA370, UV photometric). The air quality stations used the Environment SA monitoring station, which is a reference station. The quality control (QC) and quality assurance (QA) of the observed air quality data were based on the standard operating procedure published by the Presidency of Meteorology and Environment (PME).

### 2.2. Statistical Data Analysis, Modelling, and Software 

In this paper, the concentrations of three pollutants, namely, ground-level ozone (O_3_), nitrogen dioxides (NO_2_), and particulate matter of size 10 micron (PM_10_) were analysed during the COVID-19 lockdown period. To curtail the transmission of COVID-19, a lockdown was announced in the Kingdom of Saudi Arabia, which was in place from March–August 2020 [20]. The lockdown affected road traffic, working patterns, social activities, and energy consumption, which indirectly affected atmospheric pollution in urban areas. To extract the effect of lockdown on atmospheric pollution, hourly levels of O_3_, NO_2_, and PM_10_ in the months of March–August in 2020 were compared with 2019. Here, we employed two approaches:

(a) Comparing measured concentrations of 2020 with 2019 during the lockdown period, and
(b) Comparing the predicted and measured concentrations during the lockdown months in 2020 by employing a supervised machine learning approach—generalised additive model (GAM).

To extract the effect of the COVID-19 lockdown, several researchers have compared the concentrations in the pre-lockdown period with the lockdown period (e.g., [20]); however, this approach is not recommended as it does not take into account changes in the amount of emissions and meteorological conditions in different times of the year [10]. Therefore, the same months in previous years should be compared with the months during the lockdown period [10]. Some researchers have used averaged data of the previous several years, for example, the average of 2015–2019; however, due to data unavailability, this was not possible and therefore, we only used data for 2019. The model was trained using meteorological and temporal parameters for the year 2019 and then used to predict the pollutant concentrations for the lockdown period using a business-as-usual scenario [24]. 

All data analyses were performed in R programming language [25] using several of its packages: ‘openair’ [26], ‘mgcv’ [27], and lubridate [28]. ‘mgcv’ was used to develop the GAM model and predict pollutant levels for the business-as-usual (BAU) scenario. The ‘openair’ package was used to calculate correlation coefficients and root mean squared error (RMSE) values and produce various visualisations. The ‘lubridate’ package was used to edit the date and add temporal variables to the model. In the first approach, the mean concentration was calculated for the lockdown period in 2019 and 2020. Along with the mean, minimum and maximum concentrations were also calculated to show the range of the concentration. Using these values, the difference between 2019 and 2020 was calculated. 

A GAM was developed using several meteorological and temporal parameters to predict the levels of NO_2_, O_3_, and PM_10_ for the lockdown period. The model has been previously employed for such analysis by several researchers, for instance, [10,29,30]. GAM was implemented using the ‘mgcv’ package [27]. GAM is an interpretable supervised machine learning approach, which is able to study the nonlinear association between the modelled and explanatory variables. The three models are given below:

GAM (PM_10_ ~ s1 (temp) + s2 (ws) + s3 (wd) + s4 (rh) + s5 (hour) + s6 (yday) + s7 (wday)) (1)

GAM (NO_2_ ~ s1 (temp) + s2 (ws) + s3 (wd) + s4 (rh) + s5 (hour) + s6 (yday) + s7 (wday)) (2)

GAM (O_3_ ~ s1 (temp) + s2 (ws) + s3 (wd) + s4 (rh) + s5 (hour) + s6 (yday) + s7 (wday)) (3)

where ‘rh’ stands for relative humidity, ‘ws’ for wind speed, ‘wd’ for wind direction, ‘temp’ for temperature, ‘hr’ for the hour of the day, ‘wday’ for the day of the week, and ‘yday’ for the day of the year. S1 to S7 are the smooth functions of the explanatory variables. Firstly, the model was validated by splitting the 2019 hourly data into 25% randomly selected testing data and 75% randomly selected training data. The model performance was satisfactory for the cross-validated model. The model was refitted using the entire year 2019 data for training the model and then was used to predict the three pollutants for the lockdown period in the BAU scenario. 

## 3. Results

### 3.1. Comparison of the Measured Concentrations in 2019 and 2020 during the Lockdown Period

**Figure 3:**
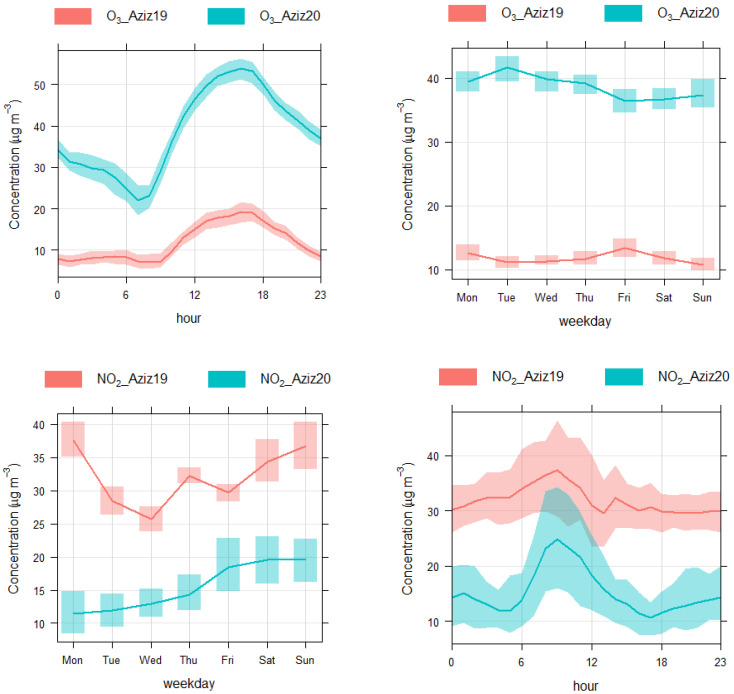
Comparing weekly and diurnal cycles of pollutant levels in 2019 and 2020 for the lockdown period at the Aziziah site in Makkah.

To calculate whether the concentrations of NO_2_, O_3_, and PM_10_ decreased or increased during the COVID-19 lockdown, the levels of these pollutants in 2019 were deducted from those in 2020; therefore, reductions in pollutant levels are shown by negative numbers, whereas increases are shown by positive numbers (Table 1). Both absolute and percent changes in pollutant levels were calculated. As an example, diurnal and weekly cycles of O_3_ and NO_2_ concentrations at the Aziziah site are depicted Figure 3, which clearly show a reduction in NO_2_ and an increase in O_3_ concentrations. However, the changes in concentrations varied from day to day and hour to hour. According to the results presented in Table 1, PM_10_ concentrations had decreased at the Aziziah, Otibiah, Shawqiah, and Umrah sites, whereas the levels had slightly increased at the Haram site. In the case of NO_2_, the concentrations decreased at the Aziziah, Otibiah, Umrah, and Haram sites, whereas due to missing data changes, those at the Shawqiah site were not calculated. In contrast, the levels of O_3_ increased at all sites, except at the Umrah site. When the levels were averaged for all sites, the levels of PM_10_ decreased by 12%, the levels of NO_2_ decreased by 58.66%, and the levels of O_3_ increased by 68.67%. 

### 3.2. Comparing Predicted and Measured Concentrations

Firstly, the models were validated to show that the fitted models performed well. For this purpose, the 2019 data were split into randomly selected training (75%) and testing data (25%). The model was fitted using the training data and validated using the independent testing data, which was not seen by the model during the training process. To assess the model performance, the two most widely used metrics, namely, root mean squared error (RMSE) and coefficient of determination (R-squared) were calculated by comparing measured and predicted concentrations of the modelled pollutant. The calculated metrics for both training (fitted model) and testing data (cross-validated) are provided in Table 2. R-squared and RMSE values showed acceptable model performance. The values for the other sites were in the same range, but slightly varied from site to site. Furthermore, the values slightly decreased for the cross-validation; however, they did not demonstrate abrupt reduction, which showed acceptable model transferability. 

**Figure 4:**
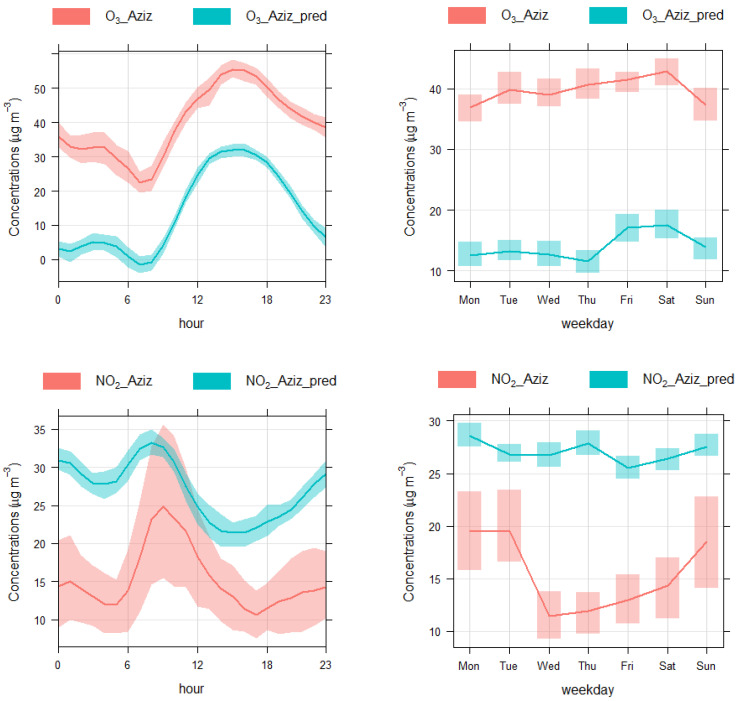
Comparing diurnal and weekly cycles of the predicted and measured concentrations during the lockdown period 2020 at the Aziziah monitoring site.

After the model validation, the models were refitted using pollutant and meteorological data along with temporal parameters for the year 2019. The trained models were reused to predict the pollutant concentrations for the BAU scenario. The concentrations predicted by the models were subtracted from the observed concentrations during the COVID-19 lockdown period. Positive difference (observed–predicted = positive difference) meant that the concentration was higher than what the model predicted; therefore, it meant the concentrations had increased during the lockdown period. In contrast, if the difference was negative between the observed and the predicted concentrations, that meant the concentration had decreased during the lockdown period. Table 3 shows the detailed results of the machine learning analysis of the three pollutants at all five monitoring sites. As an example, Figure 4 shows weekly and diurnal cycles of predicted and measured concentrations at Aziziah site. According to the model outputs, mean levels of O_3_ concentration increased by 42.45%, whereas the levels of NO_2_ and PM_10_ decreased by 12.30% and 10.75%, respectively. At the Otibiah site, the level of O_3_ increased by 19.69%, while the levels of NO_2_ and PM_10_ decreased by 4.08% and 7.90%, respectively. At the Shawqiah site, the level of O_3_ increased by 11.69%, the level of PM_10_ decreased by 6.01%, and the changes in NO_2_ were not calculated due to missing data. At the Umrah site, the levels of O_3_, NO_2_, and PM_10_ decreased by 13.93%, 35.79%, and 13.99%, respectively. Finally, at the Haram site, the level of O_3_ increased by 49.92%, while the levels of NO_2_ and PM_10_ decreased by 14.81% and 9.67%, respectively. When changes in the levels of pollutants were averaged at all five sites, it showed that overall O_3_ levels had increased by 21.96%, the levels of NO_2_ had decreased by 13.40%, and the levels of PM_10_ had decreased by 9.66%. Comparing these results with the previous section, it could be observed that the comparison of measured concentrations in 2019 with 2020 showed higher changes than those predicted by the machine learning technique. The model predictions actually removed the effect of meteorology, which affected the levels of pollutants. Hence, weather-corrected changes were moderate as compared to the changes in raw data, which is in agreement with Munir et al. [10] and Jephcote et al. [28]. 

## 4. Discussion

When the two approaches, i.e., using the measured data and using the predicted data (weather-corrected), for the lockdown period were compared, the modelling approach demonstrated less reductions in the levels of NO_2_ and PM_10_ and less increases in the levels of O_3_. However, both approaches showed similar results, i.e., increases in the level of O_3_ and reductions in the levels of NO_2_ and PM_10_ during the lockdown period. These results are in agreement with the findings of the other studies performed around the world [10,28]. Jephcote et al. [28] analysed how the levels of NO_2_, PM_2.5_, and O_3_ concentrations changed during the lockdown period and reported that the levels of both NO_2_ and PM_2.5_ had decreased, while the level of O_3_ had increased. Furthermore, Jephcote et al. [28] reported that the change in these pollutants demonstrated significant spatial variability. According to Jephcote et al. [28] pollutants experienced greater reductions at urban traffic sites than the urban background and rural sites. According to their findings, NO_2_ levels decreased by 47.9, 36.7, and 23.9%, PM_2.5_ levels decreased by 18.1, 17.3, and 2.6%, and O_3_ levels increased by 34.1, 7.4, and 0.1% at urban traffic, urban background, and rural sites, respectively. Furthermore, several other papers reported similar results, for example, [31,32,33] analysed the effect of the COVID-19 lockdown on air quality and reported that the concentrations of NO_2_ and PM_2.5_ had decreased, whereas the concentration of O_3_ had significantly increased.

The question is why O_3_ levels demonstrated positive gains while NO_2_ and PM_10_ levels demonstrated reductions during the lockdown period. It is clear that NO_2_ and PM_10_ levels are directly affected by the levels of road traffic and other emission sources, and as during the lockdown period the levels of emission decreased, they caused reductions in these pollutant concentrations. In contrast, O_3_ is a secondary pollutant, which is formed in the atmosphere by the photochemical reaction of the precursors (e.g., VOCs) and solar radiation. It is a well-known fact that O_3_ concentration is inversely proportional to NO_2_ concentration [34,35] and any reduction in NO_2_ levels causes the levels of O_3_ to increase. Therefore, when NO_2_ levels decreased during the lockdown period, it caused O_3_ levels to increase [5].

Anil and Alagha [15] studied the effect of the COVID-19 lockdown on several pollutants using air quality data from eight (8) air quality monitoring stations in the eastern province of Saudi Arabia. They considered five air pollutants in their study, which were CO, SO_2_, NO_2_, O_3_, and PM_10_. Data were obtained from four cities, namely, Jubail, Qatif, Dammam, and Al Ahsa. They reported that the levels of NO_2_ decreased at all sites during and after the lockdown periods, and the reductions ranged from 12–86% and 14–81%, respectively. The concentrations of PM_10_ (21–70%), CO (5.8–55%), and SO_2_ (8.7–30%) also decreased during the lockdown period. However, O_3_ concentrations increased ranging from 6.3% to 45%. The findings of Anil and Alagha [15] are in agreement with the current study. It is important to mention that they only used a sequential method in their study, which compared the months before, during, and after the lockdown period. This approach has its limitations and is not recommended for intervention analysis [10]. Furthermore, they did not deweathered the data, which is important to remove the effect of variation in meteorological conditions and extract the true change in air quality. Farahat et al. [36] analysed the effect of the COVID-19 lockdown on NO_2_, CO, and PM_10_ levels in three major cities in Saudi Arabia, namely, Mecca, Madinah, and Jeddah. They analysed how the levels of these pollutants had changed in these cities during the Hajj period in 2020, when the lockdown was still in place in Saudi Arabia. According to Farahat et al. [36], PM_10_ concentrations did not decrease during the lockdown period, which they attributed to frequent dust storms during the lockdown period. In contrast, the levels of NO_2_ decreased by 44%, and the levels of CO decreased by 16% during the Hajj period due to the COVID-19 lockdown restrictions. It should be noted that CO and NO_2_ are directly related with road traffic emissions and as road traffic flow was lower during the lockdown period, these two pollutants were directly affected and showed significant reductions. According to Farahat et al. [36], the levels of PM_10_ did not experience considerable change, whereas the current study showed that PM_10_ levels decreased by 58.66% according to the first approach and by 9.66% according to the second approach, when the effect of meteorological conditions was removed. Therefore, it is important to deweather air quality data for extracting the effect of the intervention.

Morsy et al. [14] analysed the effect of the COVID-19 lockdown on CO, SO_2_, NO_2_, O_3_, and PM_10_ in Makkah, Saudi Arabia using data from six air quality monitoring stations. Morsy et al. [14] compared the levels of these pollutants before and during the lockdown period and reported that the average concentrations of these pollutants decreased during the lockdown period. They demonstrated that compared with the pre-pandemic period, the concentrations of SO_2_ decreased by 26.34%, NO_2_ by 28.99%, CO by 26.24%, O_3_ by 11.62%, and PM_10_ by 30.03%. This was the only study that reported a reduction in O_3_ concentrations during the lockdown period. NO_2_ concentration is inversely proportional to the concentration of O_3_ and when NO_2_ decreases, it invariably causes O_3_ levels to increase [34]. This is what we observed in the current study as well, i.e., average O_3_ levels increased in Makkah during the lockdown period. Morsy et al. [14] used a sequential approach and did not correct the concentrations for weather variations. Furthermore, in agreement with the current study, several studies previously reported an increase in the levels of O_3_ and reductions in the levels of NO_2_ and PM_10_ during the lockdown period (e.g., [10,15,37,38,39,40]).

It was reported that the levels of pollutants (e.g., NO_2_ and PM_10_) increased again when the lockdown was relaxed [41]. This showed that the improvement in air quality was short-term and not sustainable. After the lockdown, the pollutant levels reached the previous levels or even became worse. According to Quinio and Enenkel [41], the air pollution levels recovered in three ways: (1) The pollution levels reached their levels observed before the lockdown period. This type of recovery was referred to as V-shaped recovery; (2) The pollution increased after the lockdown but still stayed lower than the levels observed before the lockdown period. This type of recovery was referred to as plateau-shaped recovery; and (3) the pollution levels after the lockdown even became worse than those observed before the lockdown. Such recovery in pollution levels was referred to as tick-shaped recovery. This means that the improvement in air quality induced by the lockdown was not sustainable, and hence there is a need for sustainable and long-term interventions to improve air quality permanently in large urban areas.

## 5. Conclusions

In this paper, the effect of the COVID-19 lockdown on the concentrations of NO_2_, O_3_, and PM_10_ was analysed using data from five air quality monitoring stations in Makkah, Saudi Arabia. Here, two approaches were employed: (1) comparing measured concentrations of the pollutants in 2019 with 2020 during the lockdown period; and (2) comparing the predictions of the machine learning models with measured pollutant concentrations during the lockdown period. The model predictions represent the weather-corrected pollutant concentrations and are generally considered more reliable. Both techniques demonstrated that the levels of NO_2_ and PM_10_ had decreased during the lockdown period due to a reduction in the levels of road traffic and other emission sources. In contrast, O_3_ concentrations had increased during the lockdown period due to the complex nature of O_3_ formation in the atmosphere. Simply, O_3_ concentration is inversely proportional to NO_2_ concentration, and therefore, as NO_2_ concentration decreased during the lockdown period, it caused the concentration of O_3_ to increase. This showed the complex interlink between different atmospheric pollutants, which should be considered when preparing an air quality management plan.

According to the first approach, the average levels of PM_10_ and NO_2_ decreased by 12% and 58.66%, respectively, whereas the level of O_3_ increased by 68.67%. According to the second approach, the O_3_ level increased by 21.96%, while the levels of NO_2_ and PM_10_ decreased by 13.40% and 9.66%, respectively. The two approaches employed in this paper resulted in slightly different amounts of changes in pollutant concentrations during the lockdown period. The first approach showed the change in raw data, whereas the second approach showed the weather-corrected change in pollutant concentrations. Therefore, the approach used for assessing the effect of the intervention is also important to take into consideration when making a decision about air quality management. Munir et al. [10] recommended two approaches for such analysis, namely, parallel and machine learning, which were used in this study. In contrast, the sequential approach, which compares the pollutant levels measured in the months just before lockdown with the concentrations measured during the lockdown period, is not recommended for such analysis as it ignores changes in emissions and meteorological conditions in different seasons of the year. The studies previously carried out in Makkah have mostly used the sequential approach, which resulted in unreliable outcomes. Therefore, this study provided an improved methodology and reliable results of the changes in pollutant concentrations during the lockdown period. The analysis suggested that the overall air quality had improved in terms of NO_2_ and PM_10_; however, the levels of O_3_ had increased. Furthermore, the improvement in air quality was not sustainable and air pollution increased again when the lockdown was relaxed. Therefore, more sustainable measures are required for permanent air quality improvement.

## Figures and Tables

**Table 1:** Percent difference (year 2020–year 2019) in the concentrations of pollutants at different monitoring sites. The negative sign shows a decrease, and the positive sign shows an increase in pollutant concentrations.

Site	Year	O_3_ (µg/m^3^)	NO_2_ (µg/m^3^)	PM_10_ (µg/m^3^)
Aziziah	2019 Mean (min, max)	22.34 (1.4, 140)	31.15 (24.4) (3.3, 340)	110.58 (7.2, 962)
	2020 Mean (min, max)	40.98 (2.3, 115.4)	22.98 (21.4) (1.7, 115)	105.68 (2, 821)
	Difference	18.64 (−89, 95)	−8.17 (−3) (−93, 97)	−4.36 (−838, 631)
	% Difference	83.44 (−401, 68)	−35.55 (−14.04) (−408, 85)	−3.95 (−757, 66)
Otibiah	2019 Mean (min, max)	42.74 (1.2, 154)	24.46 (3.1, 105.5)	123.80 (8.7, 914)
	2020 Mean (min, max)	51.75 (2.3, 148.94)	20.70 (2.7, 101.5)	109.19 (5.4, 780)
	Difference	8.03 (−97, 109)	−3.76 (−89, 91)	−14.61 (−664, 824)
	% Difference	18.78 (−227, 70)	−15.37 (−363, 86)	−11.80 (−536, 90)
Shawqiah	2019 Mean (min, max)	15.90 (4.5, 105.4)	47.66 (1.6, 305.8)	125.13 (7.3, 975)
	2020 Mean (min, max)	39.00 (3.1, 129.9)	NA	120.42 (7.3, 783)
	Difference	23.10 (−106, 80)	NA	−4.71 (−515, 896)
	% Difference	145 (−668, 76)	NA	−3.71 (−411, 92)
Umrah	2019 Mean (min, max)	55.23 (1.8, 190)	14.80 (0.4, 44.2)	118.11 (9.2, 1013)
	2020 Mean (min, max)	48.78 (1.7, 117.4)	13.83 (0, 96)	117.4 (8, 985)
	Difference	−6.45 (−76, 129)	−0.82 (−78, 33)	−0.40 (−827, 845)
	% Difference	−11.68 (−138, 68)	−5.54 (−528, 75)	−0.34 (−704, 83)
Haram	2019 Mean (min, max)	25.52 (2.1, 180)	34.98 (2.7, 108.7)	91.10 (6.7, 687)
	2020 Mean (min, max)	53.90 (2.3, 285)	34.20 (3.1, 258.6)	98.15 (3, 728)
	Difference	27.52 (−285, 128)	−0.78 (−168, 86)	7.05 (−665, 645)
	% Difference	107.82 (−943,71)	−2.2 (−480,79)	7.74 (−731, 94)

**Table 2:** Comparison of fitted and cross-validated models for predicting PM_10_, NO_2_, and O_3_ provided only for two sites i.e., the Aziziah and Haram sites.

Site	Modelled Pollutants	Fitted/Cross-Validated	R-Squared	RMSE
Aziziah	PM_10_	Fitted	0.92	6.01
		Cross-validated	0.87	7.23
	NO_2_	Fitted	0.89	6.54
		Cross-validated	0.85	6.12
	O_3_	Fitted	0.94	5.34
		Cross-validated	0.88	5.85
Haram	PM_10_	Fitted	0.93	5.97
		Cross-validated	0.91	6.23
	NO_2_	Fitted	0.90	6.07
		Cross-validated	0.89	6.32
	O_3_	Fitted	0.93	5.63
		Cross-validated	0.89	6.00

**Table 3:** Comparing observed and predicted concentrations (µg/m^3^) for the testing dataset in the business-as-usual (BAU) scenario (observed–predicted). Positive difference shows an increase whereas negative difference shows a decrease in pollutant concentrations. The values outside the parentheses are the mean values, whereas the values within the parentheses are the minimum and maximum values.

Site	O_3_ (µg/m^3^)	NO_2_ (µg/m^3^)	PM_10_ (µg/m^3^)
Aziziah_observed Mean (min, max)	42.03 (0, 115.4)	21.4 (0, 339.8)	93 (2, 821)
Aziziah_predicted Mean (max, min)	24.19 (3.2, 74.35)	24.4 (1.3, 43.45)	104.20 (11.58, 203.11)
Difference (min, max)	17.84 (−56, 70)	−3 (−43, 298)	−11.2 (−135, 726)
% difference	42.45 (−135, 165)	−12.30 (−137, 956)	−10.75 (−126, 679)
Otibiah_observed Mean (min, max)	52.64 (2, 149)	17.4 (3102)	109.37 (6, 780)
Otibiah_predicted Mean (max, min)	42.27 (1.4, 108)	18.14 (1.2, 51)	118.02 (31, 579)
Difference (min, max)	10.37 (−44, 82)	−0.74 (−27, 91)	−8.65 (−369, 654)
% difference	19.69 (−84, 156)	−4.08 (−134, 446)	−7.90 (−338, 598)
Shawqiah_observed Mean (min, max)	39.29 (1.3, 130)	NA	100 (2.1, 783)
Shawqiah_predicted Mean (max, min)	34.69 (−1, 79)	NA	106.39 (−17, 405)
Difference (min, max)	4.59 (−56, 90)	NA	−6.39 (−187, 685)
% difference	11.69 (−143, 228)	NA	−6.01 (−155, 570)
Umrah_observed Mean (min, max)	44.7 (3, 117)	11.5 (1.4, 82.3)	97 (8, 985)
Umrah _predicted Mean (max, min)	50.92 (−0.6, 115)	17.91 (−5, 43)	112.78 (39, 234)
Difference (min, max)	−6.22 (−69, 39)	−6.41 (−31, 59)	−15.78 (−148, 856)
% difference	−13.93 (−153, 87)	−35.79 (−226, 431)	−13.99 (−375, 887)
Haram_observed Mean (min, max)	53.58 (4, 285)	26.7 (3.5, 728)	81 (3, 728)
Haram_predicted Mean (max, min)	26.83 (7, 84)	31.33 (4, 85)	89.67 (0.41, 346)
Difference (min, max)	26.75 (−73, 261)	−4.64 (−69, 262)	−8.67 (−337, 599)
% difference	49.92 (−135, 488)	−14.81 (−198, 748)	−9.67 (−345, 613)

## Data Availability

Not applicable.
